# Global Bias-Corrected CORDEX Dataset at Quarter Degree Resolution

**DOI:** 10.1038/s41597-026-08060-y

**Published:** 2026-08-05

**Authors:** Fuseini Yakubu, Jürgen Böhner, Udo Schickhoff, Thomas Scholten, Shabeh ul Hasson

**Affiliations:** 1 HAREME Lab, Institute of Geography, ESRAH, Universität Hamburg, Hamburg, Germany; 2 Climate Service Center Germany (GERICS), Helmholtz-Zentrum Hereon, Hamburg, Germany; 3 Institute of Geography, ESRAH, Universität Hamburg, Hamburg, Germany; 4 Department of Geosciences, Chair of Soil Science and Geomorphology, University of Tübingen, Tübingen, Germany

## Abstract

High-resolution, bias-corrected climate data from the Coordinated Regional Downscaling Experiment (CORDEX) are essential for regional climate analysis and impact assessments. However, several existing products often rely on inconsistent adjustment methods, heterogeneous observational references, and limited spatial coverage, which hampers comparability across regions. To address this gap, we introduce GloBCORD-QD, a quasi-global dataset at 0.25° (~25 km) resolution covering the historical period (1979–2005) and future projections (2006–2099) under RCP2.6 and RCP8.5 scenarios. The dataset is generated by applying the trend-preserving ISIMIP3BASD quantile mapping approach to dynamically downscaled CORDEX-CORE simulations from four Earth System Models, using CHELSA-W5E5 as a unified observational reference across five key climate variables. Comprehensive validation demonstrates that GloBCORD-QD reduces systematic biases, increases distributional performance (Perkins Skill Score increasing from 0.66 to 0.97), and preserves climate change signals across variables, regions, and scenarios. The dataset integrates multiple CORDEX domains into a consistent quasi-global framework, enabling robust spatial comparisons while maintaining regional detail. By spanning a range of climate sensitivities across multiple models and providing methodologically consistent, high-resolution coverage, GloBCORD-QD can supports robust and comparable climate impact assessments across  various regions and sectors, contributing to improved uncertainty quantification and evidence-based climate adaptation and policy development.

## Background & Summary

High-resolution climate datasets are increasingly critical for regional climate analysis, impact assessment, and adaptation planning^1–4^. They enable improved representation of weather extremes, topography, coastlines, and other fine-scale processes that are often poorly resolved in coarse-resolution Earth System Models (ESMs*)*^5–7^. Regional Climate Models (RCMs) have therefore been widely used to dynamically downscale ESM outputs.

The Coordinated Regional Downscaling Experiment (CORDEX)^8^ established a framework for producing high-resolution regional climate projections, typically at 0.44°, 0.22°, and 0.11°. However, early CORDEX^8^ simulations at 0.44° resolution exhibited heterogeneous domain definitions and inconsistent simulation protocols, which limited comparability across regions^5,9^. The subsequent CORDEX Coordinated Output for Regional Evaluations (CORDEX-CORE)^10^ initiative addressed these limitations by introducing a unified experimental design and increasing resolution to 0.22°, enabling more consistent projections across domains^4,5,9^. In Europe, further refinement through EURO-CORDEX has demonstrated the added value of 0.11° resolution in capturing regional extremes^9^.

In parallel, global high-resolution modeling initiatives such as HighResMIP^11^ have increased the native resolution of ESMs, offering an alternative to regional downscaling^6,12–14^. Comparative studies indicate that higher-resolution RCMs generally provide improved representation of precipitation extremes, while improvements in heatwaves remain more limited^13^. Evaluations of HighResMIP^11^ and CORDEX-CORE^10^ ensembles suggest that resolution plays a key role in capturing daily precipitation distributions, with both approaches showing comparable strengths^15^. These comparisons provide important insights into the relative advantages of global high resolution modeling versus regional high-resolution modeling strategies.

For climate impact assessments, model outputs require bias adjustments to account for systematic errors^1,16,17^. While methods range from simple adjustments to advanced trend-preserving quantile mapping, challenges remain, including preserving spatial, temporal, and multivariate consistency^1,18–20^. In previous work, we developed the GloBCORD-HD^17^ dataset based on CORDEX^8^ simulations at 0.44° resolution, providing bias-adjusted climate data at ~50 km resolution using a unified methodology and a common observational reference.

Building on this foundation, we introduce GloBCORD-QD^21^, a quasi-global, bias-adjusted dataset derived from CORDEX-CORE^10^ simulations at 0.22° resolution. The dataset provides climate variables at 0.25° (~25 km) resolution for the historical period 1979–2005 and future projections (2006–2099) under RCP2.6 and RCP8.5 scenarios. Bias adjustment is performed using the trend-preserving ISIMIP3BASD v2.5^22,23^ method, with CHELSA-W5E5 v1.2^24^ as a unified observational reference. Compared to its predecessor, GloBCORD-QD^21^ incorporates higher-resolution driving simulations, improved observational reference data, with a wider CORDEX domain coverage.

By providing a consistent, high-resolution, quasi-global dataset, GloBCORD-QD^21^ can supports robust and comparable climate impact assessments across regions and sectors, including water resources, agriculture, energy, health, and ecosystems^5,22,23,25,26^.

## Methods

### Observational climate dataset

We used the CHELSA-W5E5 v1.2^24,27^ dataset (Climatologies at High Resolution for the Earth’s Land Surface Areas - WATCH Forcing Data methodology applied to ERA5 data), obtained from the ISIMIP repository (10.48364/ISIMIP.836809.3). The dataset provides daily climate data at 300 arcsec (~10 km at the equator) resolution for the period 1979–2016 and was used as the observational reference in Phase 3a of the Inter-Sectoral Impact Model Intercomparison Project (ISIMIP3a).

CHELSA-W5E5 is derived from the W5E5 v1.0^28,29^ dataset, which merges WFDE5^28,29^ over land and ERA5^30^ over oceans at 0.5° resolution. This data was first downscaled to 30 arcsec (~1 km) using the CHELSA v2.0^31^ methodology and subsequently aggregated to 300 arcsec resolution to balance spatial detail and computational efficiency^27^. The dataset provides near-global land coverage, excluding regions beyond 84°N.

**Fig. 1:**
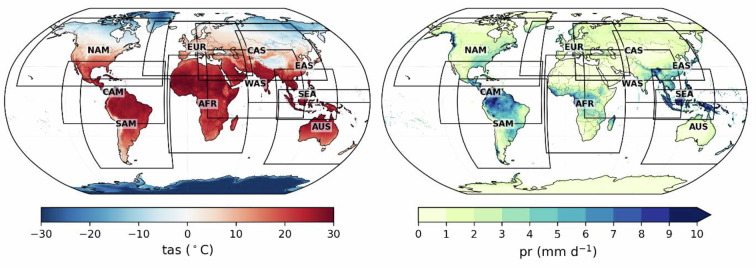
Spatial distribution of mean temperature (°C, left) and mean precipitation (mmd^−1^, right) from the CHELSA-W5E5 observational dataset over 1979–2005. Black rectangles indicate the 10 CORDEX domains used for regional climate model simulations.

At the time of this study, the dataset only included five key daily variables: mean near-surface air temperature (tas), minimum near-surface air temperature (tasmin), maximum near-surface air temperature (tasmax), total precipitation (pr), and surface downwelling shortwave radiation (rsds). Figure 1 illustrates the climatological mean temperature and precipitation for the period 1979–2005.

### CORDEX-CORE experiments

We used daily outputs from four dynamically downscaled ESMs within the CORDEX-CORE EXP-I framework (https://www.cordex.org/experiment-guidelines/cordex-core, last access: 15 May 2025). The selected ESMs are MPI-M-MPI-ESM-LR^32,33^, MPI-M-MPI-ESM-MR^32,33^, MOHC-HadGEM2-ES^34^, and NCC-NorESM1-M^35^. These models were chosen because they were among the first available in CORDEX-CORE and provide the widest coverage across regional domains (7–10 domains). For clarity, we now refer to the dynamically downscaled simulations from the CORDEX-CORE RCMs using these driving ESM as MPI-LR_CDX, MPI-MR_CDX, NorESM1_CDX, and HadGEM2_CDX, respectively.

The CORDEX-CORE simulations are typically provided at 0.22° resolution and cover the historical period (1971–2005) and future projections (2006–2100) under RCP2.6 and RCP8.5. RCP4.5 was not available for the selected ESM-RCM combinations in the CORDEX-CORE archive at the time of this study.

The selected driving ESMs also span a broad range of equilibrium climate sensitivity (ECS), capturing diverse responses to radiative forcing. MOHC-HadGEM2-ES^34^ exhibits a relatively high ECS (~4.6 K), NCC-NorESM1-M^35^ a lower ECS (~2.8 K), and the MPI models intermediate values (~3.6 K). This range is representative of CMIP5 models and ensures that the ensemble samples a wide spectrum of structural uncertainty in climate projections^17,36,37^.

These five variables (tas, tasmin, tasmax, pr, and rsds) represent some of the most important variables for climate impact assessments across sectors such as agriculture, hydrology, energy, and human health. This selection is also constrained by the CHELSA-W5E5 observational dataset, which only provided consistent coverage for these variables. While a broader set of ESMs was initially considered, limited domain availability, as well as computational and storage constraints, necessitated restricting the analysis to these four models to ensure consistency and completeness.

The CORDEX-CORE simulations collectively cover ten regional domains: Africa (AFR), Australasia (AUS), Central Asia (CAS), Central America (CAM), East Asia (EAS), Europe (EUR), North America (NAM), South America (SAM), Southeast Asia (SEA), and West Asia (WAS). Together, these domains provide quasi-global land coverage, excluding polar regions (Fig. 1). However, not all domains were available for all experiments. In particular, for the MPI-MR_CDX only 7–8 domains were available excluding the CAS and EUR.

Additional inconsistencies arise from model-specific configurations. For example, the RCP2.6 scenario was not available for MPI-MR_CDX over North American (NAM) domain. For the European domain (EUR), NorESM1_CDX, HadGEM2_CDX, MPI-MR_CDX only had data at 0.11° resolution and were therefore aggregated to 0.22° to ensure consistency.

Most simulations were produced using the REMO2015 RCM. However, MPI-MR_CDX was downscaled using multiple RCMs (RegCM4-4, RegCM4-7, and CRCM5), introducing additional variability. While this reduced RCM consistency, it also contributed to increasing the diversity of large-scale forcing conditions represented in the ensemble. Consequently, the spread in the CDX-labelled datasets reflects RCM simulations forced by their respective driving ESMs.

The CORDEX-CORE simulations were obtained from the Copernicus Climate Data Store (https://cds.climate.copernicus.eu/, last access: 15 May 2025). All variables were extracted for the full available period to match the observational reference. A summary of model-domain availability is provided in Table 1.

**Table 1:** List of four GloBCORD-QD datasets, which are prepared from dynamically downscaled four Earth System Models by either a single or multiple Regional Climate Models over distinct domains, yielding a quasi-global coverage.

No	Driving ESM and Scenarios	Run	Downscaling RCM/Version	CORDEX Domains										Variables/units
				AFR	AUS	CAM	CAS	EAS	EUR	NAM	SAM	SEA	WAS	
1	MPI-M-MPI-ESM-LR^32,33^ (Historical, RCP2.6, RCP8.5)	r1i1p1	REMO2015, v1^65^	X	X	X	X	X	X	X	X	X	X	tas(K), tasmax(K), tasmin(K), pr(Kgm^−2^s^−1^), rsds(Wm^−2^)
2	MPI-M-MPI-ESM-MR^32,33^ (Historical, RCP2.6*, RCP8.5)	r1i1p1	RegCM4-7, v0^66^	X	X	X					X	X	X	
			RegCM4-4, v0^66^					X						
			CRCM5, v1^67^							X^*^				
3	NCC-NorESM1-M^35^ (Historical, RCP2.6, RCP8.5)	r1i1p1	REMO2015, v1^65^	X	X	X	X	X	X	X	X	X	X	
4	MOHC-HadGEM2-ES^34^ (Historical, RCP2.6, RCP8.5)	r1i1p1	REMO2015, v1^65^	X	X	X	X	X	X	X	X	X	X	

Note: * means NAM is unavailable for RCP2.6.

### Preprocessing dataset

The observational dataset spans 1979–2016, from which the period 1979–2005 was selected as the historical reference, providing a 27-year overlap with the model simulations. Future projections were analyzed for 2006–2099 under RCP2.6 and RCP8.5, as several RCMs contributing to HadGEM2_CDX and MPI-MR_CDX only provide data up to 2099.

The HadGEM2_CDX simulations use a 360-day calendar with uniform 30-day months. To ensure consistency with the Gregorian calendar required by the bias adjustment method, these simulations were converted to a standard calendar. This involved removing non-standard dates (e.g., February 30) and reconstructing missing days through temporal interpolation. Specifically, missing 31st days were interpolated between the 30th of each month and the 1st of the following month for January, March, May, July, August, October, and December.

As a result, each year was adjusted to 365 days, leading to an increase of 189 days over the historical period (1979–2005) and 658 days over the future period (2006–2099). Any remaining missing timestamps were filled using linear interpolation to ensure temporal continuity and dataset completeness.

All datasets, including the observational reference, were regridded to a common global 0.25° resolution using CDO v2.4.1^38^. Temperature variables were interpolated using a bilinear scheme, while precipitation and shortwave radiation were remapped conservatively to preserve mass and energy consistency.

### Bias adjustment methodology

To generate the GloBCORD-QD^21^ dataset, we applied the Inter-Sectoral Impact Model Intercomparison Project phase 3 bias adjustment, ISIMIP3BASD v2.5^39,40^. This approach is part of the ISIMIP protocol and is widely used to prepare climate model outputs for impact assessment^17,20^. The ISIMIP3 method supports both parametric and non-parametric quantile mapping, automatically selecting the most suitable approach for each grid cell based on the Kolmogorov-Smirnov statistic^20,40,41^. This adaptive fitting ensures optimal statistical agreement between model outputs and observations prior to bias adjustment.

A key feature of ISIMIP3BASD is its trend-preserving capability, which maintains the long-term climate signal in the original model simulations. This is essential for ensuring consistency in projected climate changes and for robust representation of extreme events^39,40^. Bias adjustment was trained using the full overlapping historical period (1979–2005), corresponding to 27 years.

For shortwave radiation (rsds), the ISIMIP3BASD method produced physically unrealistic extreme values in the adjusted output when applied to CORDEX-CORE domains. This issue arises from the interaction between the bounded-variable treatment in ISIMIP3BASD and the smaller, spatially heterogeneous regional domains, leading to poorly constrained normalization. We therefore applied the ISIMIP2b^42,43^ method for rsds, which avoids this issue while remaining within the ISIMIP framework.

Following ISIMIP3 guidelines, bias adjustment for future scenarios (RCP2.6 and RCP8.5) was applied using a 31-day moving-window approach with a 1-day step, and four overlapping periods, each matching the 27-year training interval. These periods are: 1998–2024 (applied to 2006–2024), 2023–2049 (2025–2049), 2048–2074 (2050–2074), and 2073–2099 (2075–2099). This approach ensures temporal consistency while preserving evolving climate signals.

This enhances the representation of the seasonal cycle and reduces discontinuities at month boundaries. The ISIMIP3BASD v2.5 implementation is publicly available at 10.5281/zenodo.4686991, last access: 15 May 2025, along with the ISIMIP2b method at https://zenodo.org/records/1069050, last access: 15 May 2025 and the ibicus Python package used in this study at https://github.com/ecmwf-projects/ibicus, last access: 15 May 2025.

Despite its advantages, the ISIMIP3BASD method has some known limitations. Aside it still being a the univariate adjustment method, recent studies have also shown that the degree of trend preservation can vary depending on the model, variable, and climatological context^17,20,44^. Findings from Spuler *et al*. 2024, has shown that in some cases, the bias adjustment can modify projected trends substantially, with reported changes ranging up to 50–100%. Users of the dataset should therefore consider these limitations when interpreting long-term climate projections.

### Postprocessing datasets

The individual CORDEX domains were bias adjusted independently and subsequently merged to form a quasi-global dataset using an ensemble mean approach implemented in CDO. Following Lange *et al*. (2021), all variables were checked against ISIMIP3BASD-defined physical limits. Only an exceedingly small fraction of values (<0.0001%) for precipitation (pr), shortwave radiation (rsds), and temperature extremes (tasmax, tasmin) exceeded the prescribed ranges. These values were clipped to the allowed limits, with negligible impact on the overall dataset.

Due to spatial overlaps between regional domains, we further evaluated boundary consistency to identify potential discontinuities. This analysis was performed using the Normalized Root Mean Square Difference (NRMSD) approach, following Yakubu *et al*. (2025). The resulting NRMSD values were classified into four categories: Excellent, Good, Moderate, and Poor.

The evaluation indicated a high level of consistency across domain boundaries for all variables and scenarios. Most variables fall within the “Excellent” category, indicating minimal discontinuities and smooth transitions between adjacent regions. A minor exception was identified for precipitation in the MPI-LR_CDX dataset, where NRMSD values slightly exceeded the 0.5 threshold, moving it into the “Good” category.

Overall, the results demonstrate that multi-domain integration in GloBCORD-QD^21^ yields largely consistent spatial fields, supporting its suitability for both regional and global climate analyses. Detailed results of the boundary discontinuity assessment are provided in Tables S2–S5 and Figures S13–S18 in the Supplementary Material.

## Data Records

The complete bias-adjusted GloBCORD-QD^21^ dataset is publicly available at 10.25592/uhhfdm.17799. The dataset includes five daily variables: near-surface air temperature (tas), minimum temperature (tasmin), maximum temperature (tasmax), precipitation (pr), and surface downwelling shortwave radiation (rsds), for four driving ESM ensembles: MPI-LR_CDX, MPI-MR_CDX, HadGEM2_CDX, and NorESM1_CDX.

Data are provided for the historical period (1979–2005; 27 years) and for future projections (2006–2099; 94 years) under RCP2.6 and RCP8.5 scenarios. All files are distributed in netCDF format and follow the CORDEX Data Reference Syntax^45^ (DRS) convention for bias-adjusted simulations. File names follow the structure:

“VariableAdjust_GBC-25_ESM_Scenario_RCM_CHELSA-W5E5-1979-2005_day_yyyy-yyyy.nc”.

Here, “VariableAdjust” denotes the bias-adjusted variable (e.g., tasAdjust, prAdjust, rsdsAdjust). “GBC-25” refers to the Global Bias-Corrected CORDEX dataset at 0.25° resolution (GloBCORD-QD^21^), while “ESM” specifies the driving Earth System Model. “Scenario” indicates the experiment type (historical, RCP2.6, or RCP8.5), and “RCM” denotes the regional climate model(s) used in the dynamic downscaling. The term “CHELSA-W5E5-1979-2005” identifies the observational reference dataset and the period used for bias adjustment. Finally, “yyyy-yyyy” indicates the temporal coverage of each file, 1979–2005 for historical simulations and 2006–2099 for future projections. A technical summary of all variables is provided in Table 2.

**Table 2:** Technical Summary for variables in the GloBCORD-QD dataset.

Parameter	MPI-LR_CDX	MPI-MR_CDX	HadGEM2_CDX	NorESM1_CDX
Label	tasAdjust, tasminAdjust, tasmaxAdjust, prAdjust, rsdsAdjust			
Variable Name & Units	tas, tasmax, tasmin, pr, rsds K, K, K, kg m^−2^ s^−1^, Wm^−2^			
Variable Description	Near-Surface Air Temperature, Minimum Near-Surface Air Temperature, Maximum Near-Surface Air Temperature, Precipitation, Surface Downwelling Shortwave Radiation			
ECS of Driving ESM	~3.6 K		~4.6 K	~2.8 K
Historical Period	1979-2005 (27 years)			
Future Period/RCP Scenarios	2006–2099 (94 years) RCP2.6 and RCP8.5			
Spatial & Temporal Resolution	0.25° × 0.25° (~25 km) Daily			
Spatial Coverage/Grid Type	165°W to 179°E, 55°S to 76°N (Maximum) Regular latitude-longitude			
Number of Files/Format/Size	15/NetCDF/~830 Gb			
Adjustment Method	ISIMIP3BASD v2.5, ISIMIP2b			
Reference Dataset	CHELSA-W5E5 (1979–2005)			

## Technical Evaluation

### Out-of-Sample Evaluation

**Fig. 2:**
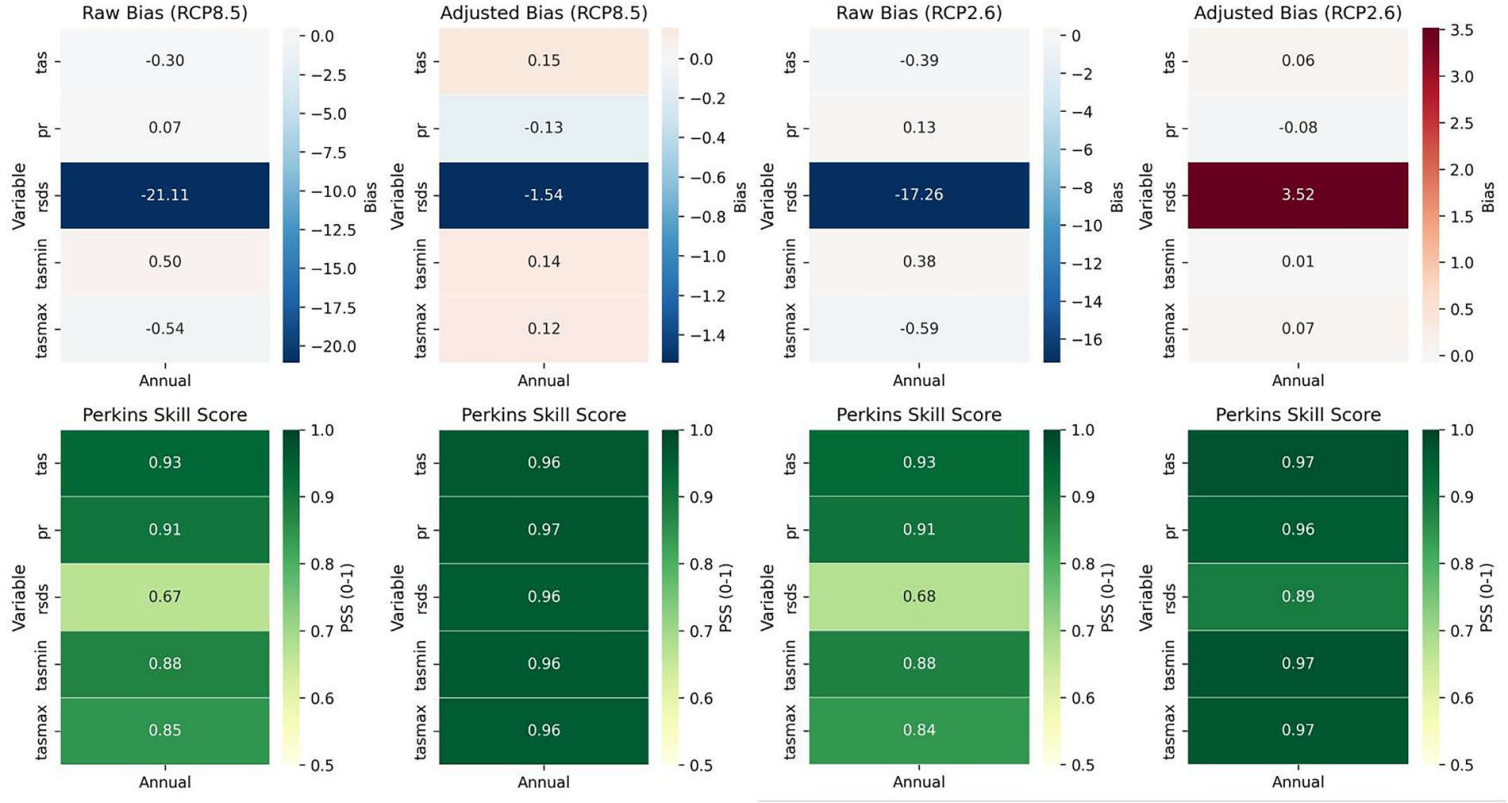
Bias and Perkins Skill Score (PSS) comparing the overlap between distribution of observed, raw model, and bias-adjusted values for 2006–2016 under RCP8.5 and RCP2.6 scenarios. The mean of seven regional domains (AFR, AUS, CAM, EAS, SAM, SEA, and WAS) was used for these analyses for MPI-LR_CDX.

To evaluate whether the bias adjustment methodology generalizes beyond the calibration period, the bias-adjusted outputs were validated against the CHELSA-W5E5 observations over the 2006–2016 period. This period lies entirely outside the historical calibration window (1979–2005) and was not seen by the bias adjustment during training. It therefore constitutes a genuine out-of-sample evaluation of future performance, using real observations as the benchmark. Results are presented for both RCP8.5 and RCP2.6 in terms of mean bias and the Perkins Skill Score (PSS), which measures the overlap between the adjusted and observed probability distributions (Fig. 2 and Figures S1–S3 in Supplementary material).

**Fig. 3:**
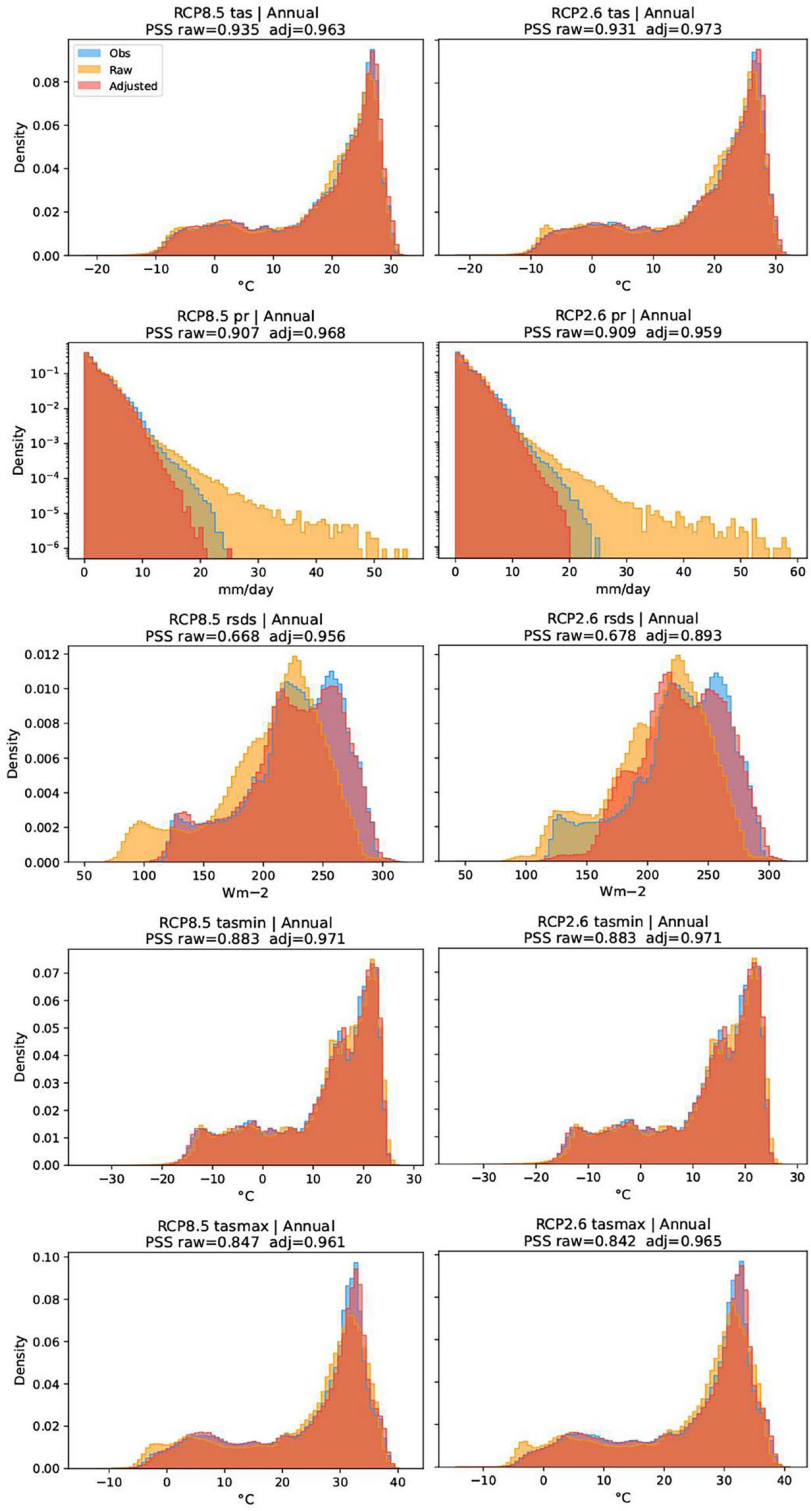
Probability Density Function (PDF) comparing the distribution of observed, raw model, and bias-adjusted values for 2006–2016 under RCP8.5 and RCP2.6 scenarios. The mean of seven regional domains (AFR, AUS, CAM, EAS, SAM, SEA, and WAS) was used for these analyses for MPI-LR_CDX.

The bias adjustment substantially reduced the systematic errors across all variables in both scenarios. Near-surface temperature variables (tas, tasmin, tasmax) exhibited small raw biases that were further reduced to near-zero after adjustment, with PSS values consistently reaching 0.89–0.97. Precipitation showed similarly strong improvement, with PSS increasing from 0.91 to approximately 0.97. The most pronounced improvement was found for surface downwelling shortwave radiation (rsds), which carried the largest raw bias (up to −17 to −25W m^−2^ under both scenarios except for MPI-MR_CDX), reduced to 3.52 and −1.54 W m^−2^ respectively after adjustment, with PSS improving from approximately 0.67–0.68 to 0.89–0.96. MPI-MR_CDX (Figure S1) showed a slightly lower bias across all models for rsds. The PDF distributions (Fig. 3 and Figures S4–S6 in Supplementary material) show the adjusted output closely tracks observations across the full distribution for all variables. Overall, the consistently high PSS values, substantially reduced biases across an entirely independent validation period and improvement in the PDF distributions demonstrate that the bias adjustment methodology generalizes robustly beyond the calibration period, providing confidence in its application to future projection data.

We also recognize that the high PSS values obtained during the independent validation period (2006–2016) do not, by themselves, rule out the possibility that the 27-year calibration period (1979–2005) sampled a particular phase of internal climate variability. To evaluate the sensitivity of the bias adjustment to the choice of calibration period, we conducted additional validation experiments using alternative calibration-validation splits: 1986–2005 for calibration with 1979–1985 for validation, and 1979–1998 for calibration with 1999–2005 for validation. These periods represent different samples of internal variability. For each experiment, the bias-adjustment transfer functions were re-estimated independently (for tas and pr), and the resulting adjusted climate statistics (Bias, Correlation, MAE, RMSE and STD_ratio) were compared. The results confirmed the robustness of the ISIMIP3BASD protocol with respect to different realizations of internal variability. Validation statistics for these experiments are provided in Table S1 of the Supplementary Material.

It should be noted that the 2006–2016 validation period represents the near-term future, during which differences in external forcing between RCP2.6 and RCP8.5 remain relatively small and the climate state has not yet diverged substantially from that of the calibration period. Consequently, the robustness of the bias adjustment under the markedly different climate conditions projected for the mid- to late twenty-first century cannot be directly verified against observations and remains a general limitation of bias-adjusted climate projection datasets. Users applying GloBCORD-QD under high-end warming scenarios should therefore be aware of this limitation.

### Spatial evaluation

The evaluation framework integrates spatial, temporal, and statistical perspectives, following established best practices for post bias-adjustment assessment^46,47^. Historical biases were calculated as the differences between simulated (raw or adjusted) and observed climatologies over the 1979–2005 period.

**Fig. 4:**
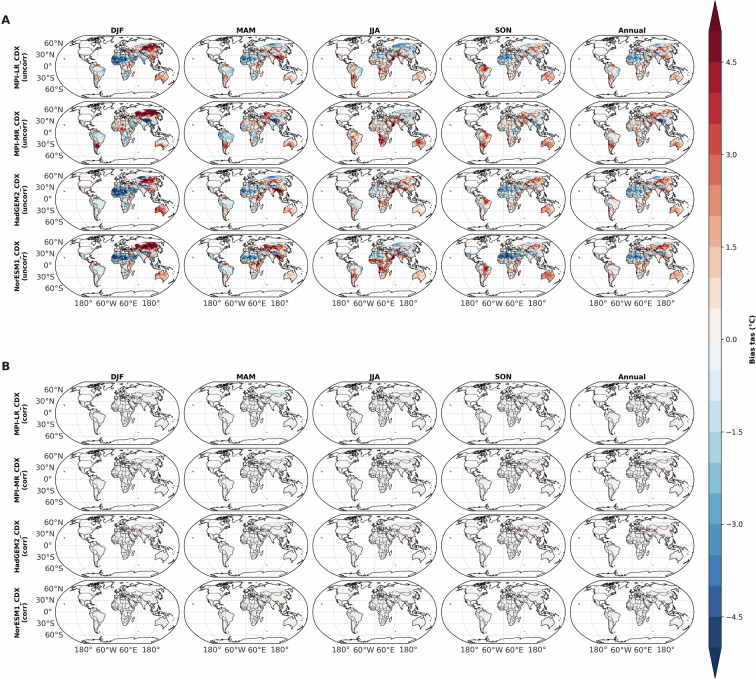
Biases in mean seasonal and annual near-surface air temperature (tas, in °C) for the historical period (1979–2005) against CHELSA-W5E5. The top four rows (**A**) correspond to each of the driving ESMs for unadjusted, and the bottom four rows (**B**) to bias-adjusted datasets.

Figure 4 illustrates temperature biases, with values ranging approximately within ±5 °C for the raw simulations. The raw CORDEX datasets exhibited systematic cold biases across large regions, including the Sahara, equatorial zones, and the Himalayas, with particularly strong deviations in the MPI-MR_CDX. Such cold biases are commonly reported in regional climate models and are often linked to limitations in representing surface processes in arid and mountainous regions^48,49^. In addition, warm biases are evident in East Asia, Australia, and southern South America. Similar pattern can be seen for minimum and maximum temperatures in Figures S8 and S9 in the supplementary material.

Following bias adjustment, temperature biases are reduced across all regions, falling within ±0.3 °C. This level of improvement is consistent with the performance reported for advanced quantile mapping techniques^16,50^. Seasonal analysis indicates that cold biases are most pronounced during winter in continental interiors, a known characteristic of CORDEX simulations^50,51^.

**Fig. 5:**
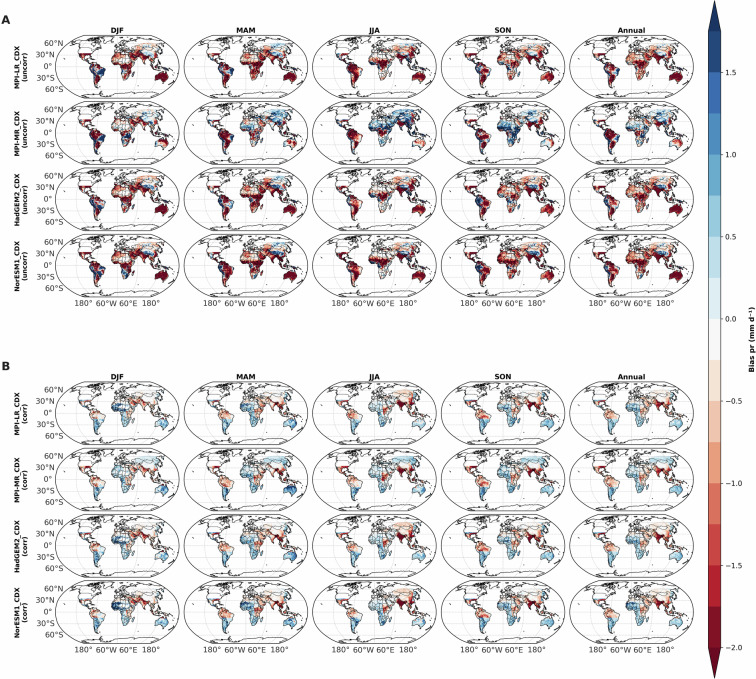
Same as Fig. 4 but for precipitation (pr, in mm/d).

Precipitation biases (Fig. 5) show initial deviations of up to ±3 mm/day, with widespread dry biases across most models except MPI-MR_CDX. Positive biases are particularly evident in coastal regions, reflecting known challenges in representing land-sea interactions and orographic processes^52,53^. Negative biases are observed over tropical regions such as the Amazon, Central and Southern Africa, and parts of West Asia, consistent with limitations in simulating convection and land-atmosphere interactions^54,55^.

After adjustment, precipitation biases are reduced, with most regions showing biases within ±0.5 mm/day. Residual biases remain spatially structured, with slight dry biases in parts of Africa, South Asia, Australia, and East Asia, while other regions exhibit minor wet biases.

Figure S7 presents biases in surface downwelling shortwave radiation (rsds), with initial deviations of up to ±50 W/m². Most models exhibit systematic underestimation, particularly during boreal summer (JJA), likely due to overestimated cloud cover and aerosol effects^56^. In contrast, MPI-MR_CDX shows positive biases (~25–45 W/m²), reflecting differences arising from the use of multiple RCMs.

The bias adjustment improved rsds simulations, reducing discrepancies to within approximately ±2 W/m² across most regions and seasons. While some residual biases persist, the overall performance represents a significant improvement relative to the raw model output.

Overall, the results demonstrate that the applied bias adjustment framework effectively reduces systematic differences across all variables and domains while preserving physically meaningful spatial and seasonal patterns. The residuals are partly linked to RCM, variable and the domain characteristics.

### Temporal evaluation

For all analyses of time series, seasonal cycles, and global mean statistics, a common spatial mask was applied to ensure consistent comparisons across datasets. This mask corresponds to the seven CORDEX-CORE domains available in MPI-MR_CDX under RCP2.6 (AFR, AUS, CAM, EAS, SAM, SEA, and WAS) even though the other datasets (MPI-LR_CDX, HadGEM2_CDX, and NorESM1_CDX) inherently cover a larger spatial extent. Consequently, statistics derived using this mask do not fully represent the complete quasi-global coverage of the other datasets and should be interpreted accordingly.

**Fig. 6:**
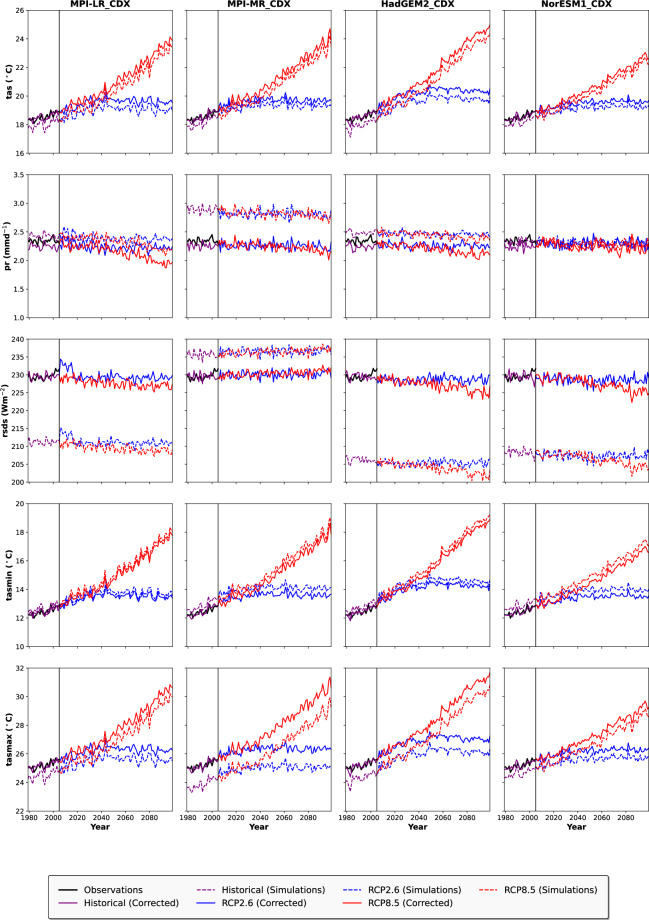
Yearly time series for tas, tasmin, tasmax, pr, and rsds considering the mean of seven regional domains of AFR, AUS, CAM, EAS, SAM, SEA, and WAS for all four ESMs. Each column represents each dataset from a particular downscaled ESM, whereas each row presents individual variables from a particular dataset. The black line divides the historical period 1979–2005 from the future period 2006–2099. Historical observations and model simulations are shown in black and magenta, whereas future projections for RCP2.6 and RCP8.5 are presented in blue and red, respectively.

Figure 6 presents the annual mean temperature (tas) time series for the period 1979–2099. A clear warming trend is evident during the historical period, followed by a pronounced divergence between scenarios after mid-century. Under RCP8.5, temperatures increase sharply, exceeding 4 °C in some models by the end of the century, whereas RCP2.6 exhibits stabilization after mid-century. HadGEM2_CDX projects the strongest warming, consistent with the relatively high equilibrium climate sensitivity (~4.6 K) of its driving ESM, while NorESM1_CDX also shows the weakest warming representing the influence from its driving ESM (~2.8 K ECS). This relationship between ECS and projected warming is consistent with findings from other climate model intercomparison studies^55,56^.

**Fig. 7:**
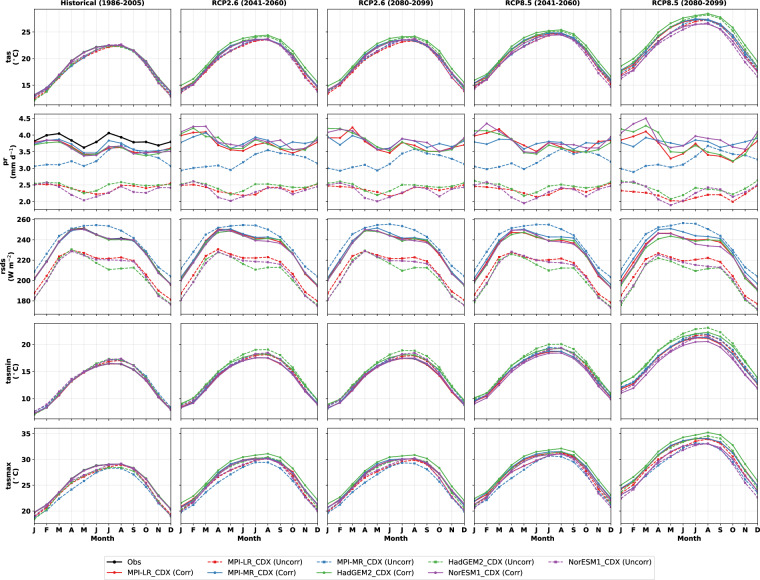
Climatological annual cycle of tas, pr, rsds, and tasmin, tasmax considering the mean of seven regional domains of AFR, AUS, CAM, EAS, SAM, SEA, and WAS for the historical period (1986–2005), RCP2.6 and RCP8.5 for two futures, Mid-century (2041–2060), and Late-century (2080–2099).

The seasonal cycle of temperature (Fig. 7) shows strong agreement with observations, with peaks in summer and minima in winter. The models reproduce the observed seasonal pattern well, although slight differences in amplitude are evident across models and scenarios. Projected warming occurs in all months, with larger increases during summer and in late-century periods, particularly under RCP8.5.

In contrast to temperature, precipitation (pr) exhibits relatively weak long-term trends (Fig. 6), with interannual variability dominating the signal. Some models show slight decreases over time, but these changes are small relative to natural variability. This behavior is typical of precipitation projections, where variability often masks long-term trends^57,58^. The seasonal cycle (Fig. 7) is well captured, with peak rainfall during wet seasons and reduced precipitation during dry periods. However, there is a small but consistent underestimation of mean precipitation (~0.05–0.2 mm d^−2^) in the bias-adjusted historical period in the seasonal cycle. This small underestimation reflects conservative adjustment behaviour and is a limitation of the ISIMIP3BASD methodology. Future precipitation changes remain modest and model dependent. Some models indicate increases during wet seasons, while others show slight declines, and no consistent shift in seasonal patterns is observed across the ensemble.

Surface downwelling shortwave radiation (rsds) shows relatively stable long-term behavior, with only minor scenario-dependent changes (Fig. 6). Variability is comparable to that of precipitation, with no strong long-term trend. MPI-MR_CDX exhibits slightly higher values compared to the other models, likely reflecting differences arising from its use of multiple RCMs. The seasonal cycle of rsds (Fig. 7) is strongly driven by solar geometry and is well reproduced by the models. While small amplitude differences are present, projected changes remain limited, with only minor increases or decreases depending on model and season. These variations may be associated with changes in cloud cover or atmospheric transparency.

## Extra Insights

### Climate Signal

The effectiveness of a trend-preserving bias adjustment methods is not only determined by their ability to reduce systematic biases, but also by their capacity to preserve the original climate change signals of the driving models. Trend preservation is therefore a critical requirement for bias adjustment in climate impact studies^39,57^. To assess this, we evaluated the difference in projected change signals (Δ Diff) between raw and bias-adjusted datasets.

The change signal was defined as the difference between future periods and a historical baseline (1986–2005). Two future time slices were considered: mid-century (2041–2060) and late-century (2080–2099), under both RCP2.6 and RCP8.5 scenarios, following IPCC AR6 conventions^59^. The late-century period slightly differs from the standard 2081–2100 interval due to future data ending in 2099.

The Δ Diff metric quantifies the extent to which bias adjustment modifies the original climate signal. Values close to zero indicate successful trend preservation, while larger deviations suggest that the adjustment process alters the underlying model response. This approach follows established methodologies for evaluating trend-preserving bias adjustment^40^.

**Fig. 8:**
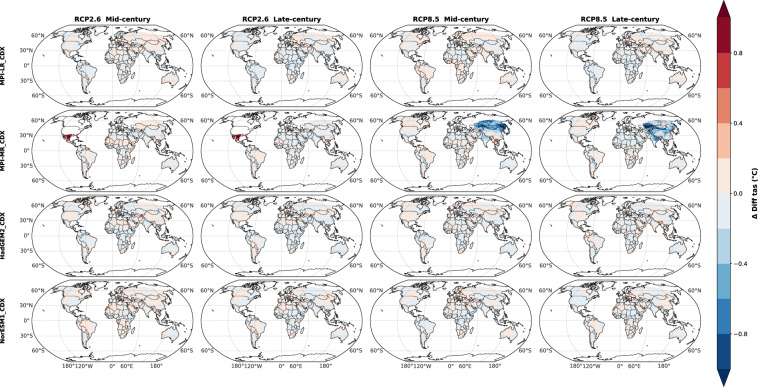
Difference in tas change signal (Δ Diff, °C) for the RCP2.6 and RCP8.5 scenarios for the Mid-century (2041–2060) and Late-century (2080–2099).

**Fig. 9:**
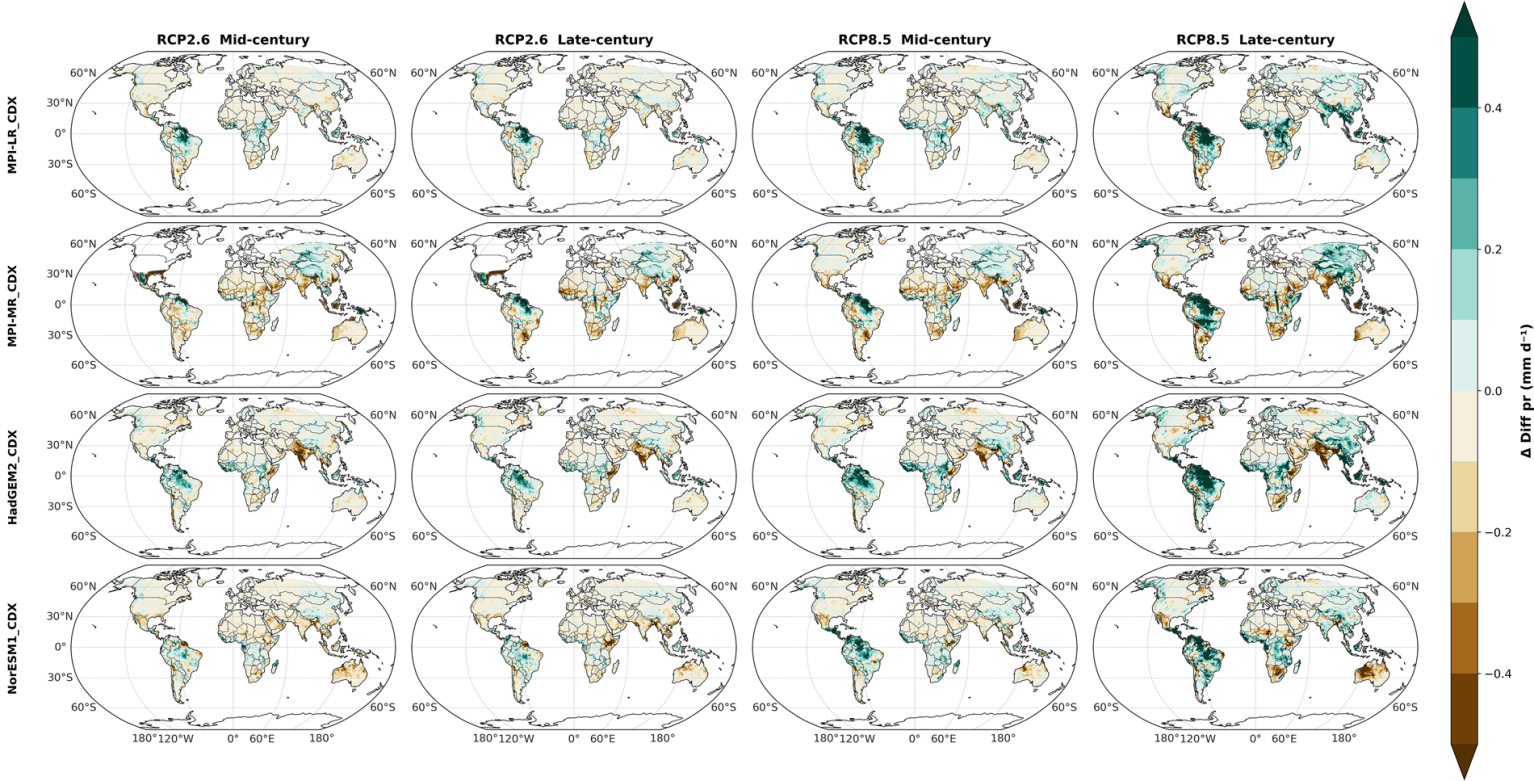
Same as Fig. 8, but for pr (mm/d).

Overall, the results indicate strong trend preservation across all variables, models, and scenarios (Figs. 8 and 9; Figures S10–S12). Δ Diff values remain small, generally within ±0.6 °C for temperature, ±0.4 mm/day for precipitation, and ±2.5 W/m² for shortwave radiation. These results demonstrate that the bias adjustment effectively reduces systematic errors while largely maintaining the original climate change signals^60,61^.

For temperature (tas), Δ Diff values are consistently low (typically within ±0.6 °C), indicating that projected warming trends are well preserved. No systematic spatial patterns of trend distortion are evident. Isolated deviations (up to ~0.8 °C), such as over parts of Central America and East Asia in MPI-MR_CDX under RCP2.6 and RCP8.5 respectively, are spatially limited and appear non-systematic. A plausible interpretation is that, in these specific domains, the combination of strong distributional changes in these RCM and the extrapolation behaviour of the bias adjustment leads to slight distortions in the adjusted change signal.

Precipitation also exhibits robust trend preservation, with Δ Diff values generally within ±0.4 mm/day. The spatial distribution of projected changes remains consistent across climate regimes. Some increased variability is observed in regions such as northeastern South America and parts of Southeast Asia, but these deviations are small and spatially scattered, indicating no systematic artifacts introduced by the bias adjustment.

Shortwave radiation (rsds) shows similarly small deviations, with most values within ±2.5 W/m². In many regions, differences are even smaller (typically between −1 and +0.5 W/m²), indicating minimal alteration of radiation trends. Slightly larger deviations (~1–3 W/m²) are observed in some regions, including northern North America, Europe, and Central Asia in MPI-LR_CDX, as well as localized areas in other models.

These deviations may be attributed to the use of the ISIMIP2b method for rsds, which was applied to avoid artifacts produced by ISIMIP3BASD. As this method is less explicitly trend-preserving, small modifications of the signal are expected, particularly in regions where the original model exhibited strong biases. Nonetheless, the overall preservation of radiation trends remains high, ensuring that key processes such as radiative forcing and cloud-radiation interactions are largely maintained.

A localized exception to the generally robust performance is observed in the MPI-MR_CDX dataset. In the East Asia (EAS) domain, downscaled using RegCM4-4, a dampened warming signal is evident under RCP8.5. In contrast, the Central America (CAM) domain, downscaled using RegCM4-7, shows elevated positive Δ Diff values under RCP2.6. These anomalies are spatially confined and are not present in the other datasets.

We attribute these deviations to the combined effects of RCM-specific behavior and methodological limitations. The RegCM4 configurations produce distributional characteristics that differ from other RCMs under the same forcing conditions. Furthermore, as noted in previous studies, even trend-preserving bias adjustment methods may alter change signals when future climate conditions extend beyond the calibration range or when distributional assumptions are not fully satisfied^20^.

Overall, the results confirm that the applied bias adjustment approach preserves climate change signals to a high degree of accuracy, with only minor, localized deviations.

### Data distribution

**Fig. 10:**
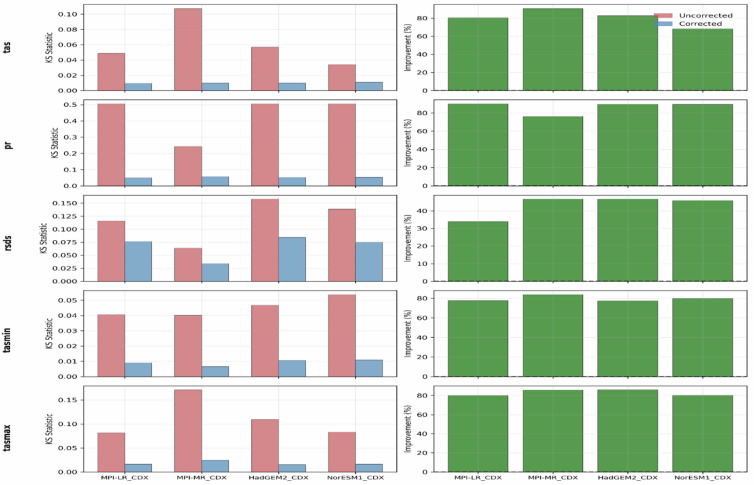
The Kolmogorov-Smirnov statistics (left column) with percentage improvement (right column) for the adjusted and raw historical (1979–2005) tas, pr, rsds, tasmin, and tasmax for the four CORDEX ensembles of MPI-LR_CDX, MPI-MR_CDX, HadGEM2_CDX, and NorESM1_CDX, considering the average of seven domains (AFR, AUS, CAM, EAS, SAM, SEA, and WAS).

To further evaluate distributional performance, we applied the Kolmogorov-Smirnov (KS)^62^ statistic as a measure of agreement between model outputs and observations (Fig. 10). The KS statistic is widely used for assessing distributional differences in climate data, as it quantifies the maximum distance between the empirical cumulative distribution functions of two datasets^45,61^. In this study, it was computed using daily values, with the CHELSA-W5E5 serving as the observational reference.

The KS results reveal distributional discrepancies in the unadjusted model outputs across most variables and models. For temperature and precipitation variables (tas, tasmin, tasmax, and pr), KS values for the raw data range approximately between 0.04 and 0.5, indicating slight deviations from the observed distributions.

Following bias adjustment, KS values decrease consistently across all models and variables, demonstrating a significant improvement in distributional agreement. However, small residual differences remain, indicating that while the bias adjustment substantially reduces discrepancies, perfect alignment is not achieved.

The relative improvement in distributional agreement exceeds 75% for most variable-model combinations, as shown in Fig. 10. For rsds, improvements are more moderate, typically ranging between ~35% and ~58%. These results are consistent with previous studies evaluating advanced bias adjustment methods and confirm the effectiveness of the applied approach^17,19,63^.

Overall, the KS-based assessment demonstrates that the bias adjustment framework significantly improves the representation of the variable distributions while retaining small, localized residual differences.

GloBCORD-QD^21^ offers several key advantages for climate impact assessments and research. First, at a spatial resolution of 0.25° (~25 km), it provides finer detail than most global ESMs (typically 50–200 km) and many existing bias-adjusted datasets. Second, the dataset is based on dynamically downscaled RCM outputs that explicitly resolve regional physical processes, including topographic effects, land-surface interactions, and mesoscale phenomena. This approach enables a more realistic representation of local climate drivers compared to purely statistical downscaling methods. The effectiveness of our spatial validation approach is consistent with established methods for evaluating dynamically downscaled climate data^14,17,50,64^. Third, GloBCORD-QD^21^ is among the few quasi-global climate datasets derived from CORDEX-CORE^10^ RCMs using standardized protocols, ensuring spatial consistency across domains and enabling robust cross-regional comparisons.

Despite these advantages, several limitations should be considered when using the dataset. First, like all bias-adjusted products, GloBCORD-QD^21^ inherits potential biases and uncertainties from its reference dataset. As a result, its overall accuracy is fundamentally constrained by the quality and representativeness of the underlying observational data. Second, it uses ensemble means across overlapping CORDEX domains; therefore, data in these overlapping regions represents a combination of multiple RCM outputs. Third, due to the spatial coverage limitations inherent to CORDEX^8^ simulations, GloBCORD-QD^21^ only provides a quasi-global coverage as compared to other global datasets. Fourth, we acknowledge that the relative short calibration period (27 years) does not fully exclude a contribution from the internal variability to the estimated climatology. Finally, the merging of different RCMs and domains may introduce spatial discontinuities at domain boundaries. Although our analysis indicates that transitions at these boundaries are seamless (see Supplementary Figures S13 and S14, Tables S2–S5 and Figures S15–S18), caution is still advised over these areas. In particular, users should be extra cautious when using MPI-MR_CDX data over the EAS domain under RCP8.5 and the CAM domain under RCP2.6, as the projected change signals in these domain-RCM combinations contain additional uncertainty compared to the rest of the dataset.

GloBCORD-QD^21^ represents an important advancement in high-resolution climate datasets for impact assessments. By combining 0.25° spatial resolution with dynamically downscaled CORDEX-CORE simulations and a robust bias adjustment framework, the dataset provides quasi-global coverage with enhanced regional detail.

Validation results demonstrate that the ISIMIP3BASD v2.5 bias adjustment approach effectively reduces systematic biases while preserving climate change signals across multiple variables, emission scenarios, and time periods. These findings are consistent with previous evaluations of trend-preserving quantile mapping methods and confirm their suitability for climate impact applications.

The dataset spans a broad range of climate sensitivities (ECS ~2.8 K to ~4.6 K) across four dynamically downscaled ESMs, providing a representative range of model responses for uncertainty assessment. This diversity supports more robust analysis of climate risks and impacts.

With open access and comprehensive documentation, GloBCORD-QD^21^ addresses a key need for consistent, high-resolution, bias-adjusted climate data. It provides a valuable resource for climate research and supports evidence-based decision-making across sectors including water resources, agriculture, energy, health, and ecosystems.

## Supplementary information

Supplementary Information

## Data Availability

The dataset is available at the Universität Hamburg’s Zentrum. für Nachhaltiges Forschungsdatenmanagement using 10.25592/uhhfdm.17799.

## Code availability

The regridding of climate simulations to the reference grid was performed in Climate Data Operator (CDO), which can be obtained from the Max Planck Institute of Meteorology using the link https://code.mpimet.mpg.de/projects/cdo. The bias correction algorithm of ISIMIP3BASD is publicly available and can be obtained from the repositories (10.5281/zenodo.4686991, last access: 15 May 2025) for ISIMIP3BASD and (https://zenodo.org/records/1069050, last access: 15 May 2025) for ISIMIP2b. The ibicus Python package used in the bias correction is freely available at (https://github.com/ecmwf-projects/ibicus, last access: 15 May 2025). Figures are plotted in Python using matplotlib; the correction scripts used is available at 10.25592/uhhfdm.17395.
