# Supplementary material for: Global Bias-Corrected CORDEX Dataset at Quarter Degree Resolution
Source: Sci Data. 2026 Aug 5;13:1135. doi: 10.1038/s41597-026-08060-y (PMC13443925; doi:10.1038/s41597-026-08060-y)
Supplement: Supplementary file 1 [file 41597_2026_8060_MOESM1_ESM.docx]

**Supplementary Information**

Figure S1. Bias and Perkins Skill Score (PSS) comparing the overlap between distribution of observed, raw model, and bias-adjusted values for 2006-2016 under RCP8.5 and RCP2.6 scenarios. The mean of seven regional domains (AFR, AUS, CAM, EAS, SAM, SEA, and WAS) was used for these analyses for MPI-MR_CDX.

Figure S2. Same as Figure S1, but for HadGEM2_CDX.

Figure S3. Same as Figure S1, but for NorESM1_CDX.

Table S1. Validation statistics for alternative calibration-validation period splits used to assess the sensitivity of the ISIMIP3BASD bias adjustment to internal climate variability.

| Model | Variable | Bias | MAE | RMSE | Corr | STD_ratio | Calibration Period | validation Period |
| --- | --- | --- | --- | --- | --- | --- | --- | --- |
| MPI-LR_CDX | tas/K | 0.10 | 0.50 | 0.66 | 0.89 | 0.99 | 1986-2005 | 1979-1985 |
| MPI-LR_CDX | tas/K | 0.22 | 0.56 | 0.72 | 0.88 | 1.03 | 1979-1998 | 1999-2005 |
| MPI-LR_CDX | pr/mm/d | 0.10 | 1.31 | 1.81 | 0.44 | 0.90 | 1986-2005 | 1979-1985 |
| MPI-LR_CDX | pr/mm/d | -0.08 | 1.28 | 2.04 | 0.82 | 1.00 | 1979-1998 | 1999-2005 |
| MPI-MR_CDX | tas/K | 0.32 | 0.68 | 0.86 | 0.90 | 1.09 | 1986-2005 | 1979-1985 |
| MPI-MR_CDX | tas/K | 0.20 | 0.60 | 0.76 | 0.92 | 1.05 | 1979-1998 | 1999-2005 |
| MPI-MR_CDX | pr/mm/d | -0.06 | 1.28 | 1.80 | 0.84 | 0.92 | 1986-2005 | 1979-1985 |
| MPI-MR_CDX | pr/mm/d | 0.24 | 2.23 | 3.57 | 0.74 | 1.04 | 1979-1998 | 1999-2005 |
| HadGEM2_CDX | tas/K | 0.04 | 0.39 | 0.48 | 0.86 | 0.93 | 1986-2005 | 1979-1985 |
| HadGEM2_CDX | tas/K | 0.05 | 0.41 | 0.50 | 0.85 | 0.98 | 1979-1998 | 1999-2005 |
| HadGEM2_CDX | pr/mm/d | -0.17 | 1.02 | 1.77 | 0.79 | 0.85 | 1986-2005 | 1979-1985 |
| HadGEM2_CDX | pr/mm/d | 0.09 | 1.32 | 2.02 | 0.85 | 1.05 | 1979-1998 | 1999-2005 |
| NorESM1_CDX | tas/K | 0.07 | 0.49 | 0.66 | 0.85 | 0.96 | 1986-2005 | 1979-1985 |
| NorESM1_CDX | tas/K | 0.17 | 0.53 | 0.70 | 0.85 | 1.04 | 1979-1998 | 1999-2005 |
| NorESM1_CDX | pr/mm/d | -0.10 | 1.10 | 1.74 | 0.90 | 1.01 | 1986-2005 | 1979-1985 |
| NorESM1_CDX | pr/mm/d | -0.19 | 2.08 | 2.82 | 0.85 | 1.03 | 1979-1998 | 1999-2005 |

Figure S4. Probability Density Function (PDF) comparing the distribution of observed, raw model, and bias-adjusted values for 2006-2016 under RCP8.5 and RCP2.6 scenarios. The mean of seven regional domains (AFR, AUS, CAM, EAS, SAM, SEA, and WAS) was used for these analyses for MPI-MR_CDX.

Figure S5. Same as Figure S4, but for HadGEM2_CDX.

Figure 6. Same as Figure S4, but for NorESM1_CDX.

Figure S7 . Biases in mean seasonal and annual Shortwave radiation (rsds, in W/m2) for the historical period (1979-2005) against CHELSA-W5E5. The top four rows correspond to each of the driving ESMs for uncorrected, and the bottom four rows to bias-corrected datasets.

Figure S8. Same as in Figure S1, but for Maximum Temperature(tasmax, in °C)

Figure S9. Same as in Figure S1, but for Minimum Temperature(tasmin, in °C)

Figure S10. Difference in rsds change signal (Δ Diff, W/m^2^) for the RCP2.6 and RCP8.5 scenarios for the Mid-century (2041-2060) and Late-century (2080-2099).

Figure S11. Same as Figure S4, but for tasmax (°C)

Figure S12. Same as Figure S4, but for tasmin (°C)

# Boundary Discontinuities in GloBCORD-QD

Following Yakubu et al., 2025^1^, we used the Normalized Root Mean Square Difference (NRMSD)^2^ based methodology to quantify and evaluate discontinuities across the multiple climate variables, scenarios, and domain interfaces. This approach objectively quantifies boundary discontinuities, highlighting areas of transition among domains.

## Boundary Detection and Construction

For each domain, we first calculated the RMSD between each boundary grid cell and its neighboring grid cell from the overlapping domains for all overlapping boundaries (eqn. 1). Then, the grid cell-based RMSD was averaged over each domain. The domain-averaged RMSD was then normalized by the averaged Standard Deviations (eqn. 2) of both overlapping domains, i.e., NRMSD (eqn. 3). Standard deviation for each domain was calculated from the pooled data from the boundary grid cells that participated in RMSD calculation.

Figure S13. A global map showing the regional climate model domain overlaps by the number of domains. The colours indicate the number of overlapping domains.

Figure S14. a). Same as Figure S13, but showing boundaries between overlapping RCMs. b). A map showing the boundary grid cell pairs with possible discontinuities in GloBCORD-QD.

$RMSD=\sqrt{\frac{1}{T}\sum_{t=1}^{T} \left[ X_{d_{1}}\left( t \right)-X_{d_{2}}\left( t \right) \right]^{2}}$ *.............. eqn.1*

*Sd*$=\sqrt{\frac{1}{N-1}\sum_{i=1}^{N} \left( X_{d_{n}}\left( t \right)- \bar{X}_{d_{n}} \right)^{2}}$ .............. eqn.2

$NRMSD=\frac{RMSD}{\sqrt{(\frac{Sd₁^{2} + Sd₂^{2}}{2})}}$	.............. eqn.3

Where:

- T = Total n*umber of time steps*
- t = Time *index*
- $X_{d_{n}}\left( t \right)$ = Spatial mean (averaged across all boundary cells on domain n side) at each time step t
- Sd = Standard deviation of Data points
- $\bar{X}$= Mean of data
- N = Total number of boundary segments/grid pairs
- n = Domain side (1 or 2)

We grouped the NRMSD into four categories of excellent, good, moderate, and poor based on the following criteria:

- NRMSD < 0.5 (Excellent): discontinuity is less than half the natural climate variability
- NRMSD = 0.5-1.0 (Good): discontinuity comparable to natural variability
- NRMSD = 1.0-2.0 (Moderate): discontinuity slightly exceeds natural variability
- NRMSD > 2.0 (Poor): discontinuity significantly exceeds natural variability

# Results

The domain boundary discontinuities across all variables and RCP scenarios indicate that the GloBCORD-QD^3^ dataset exhibits seamless multi-domain integration. Except for precipitation in the MPI-LR_CDX data, which slightly exceeded the 0.5 thresholds for mean NRMSD, all other datasets, variables, and scenarios in the GloBCORD-QD^3^ data fell within the excellent category. This means the data shows significant seamless transitions across domain boundaries without discontinuities, promoting the suitability of the data for regional/global climate analysis.

Table S2. Table showing Discontinuity Statistic for MPI-LR_CDX

| **Variable** | **Scenario** | **Mean**  **RMSD** | **Std**  **RMSD** | **Mean**  **NRMSD** | **Std**  **NRMSD** | **Quality** | **Grid Cell**  **Count** |
| --- | --- | --- | --- | --- | --- | --- | --- |
| pr | historical | 0.000 | 0.000 | 0.512 | 0.189 | GOOD | 3092 |
| pr | rcp26 | 0.000 | 0.000 | 0.533 | 0.208 | GOOD | 3092 |
| pr | rcp85 | 0.000 | 0.000 | 0.536 | 0.199 | GOOD | 3092 |
| rsds | historical | 26.458 | 16.975 | 0.230 | 0.198 | EXCELLENT | 3092 |
| rsds | rcp26 | 26.626 | 17.232 | 0.303 | 0.200 | EXCELLENT | 3092 |
| rsds | rcp85 | 25.999 | 16.896 | 0.295 | 0.196 | EXCELLENT | 3092 |
| tas | historical | 1.194 | 1.155 | 0.203 | 0.392 | EXCELLENT | 3092 |
| tas | rcp26 | 1.269 | 1.185 | 0.209 | 0.377 | EXCELLENT | 3092 |
| tas | rcp85 | 1.196 | 1.134 | 0.177 | 0.271 | EXCELLENT | 3092 |
| tasmax | historical | 1.388 | 1.127 | 0.213 | 0.279 | EXCELLENT | 3092 |
| tasmax | rcp26 | 1.468 | 1.160 | 0.219 | 0.272 | EXCELLENT | 3092 |
| tasmax | rcp85 | 1.406 | 1.118 | 0.195 | 0.232 | EXCELLENT | 3092 |
| tasmin | historical | 1.360 | 1.190 | 0.206 | 0.306 | EXCELLENT | 3092 |
| tasmin | rcp26 | 1.419 | 1.218 | 0.212 | 0.301 | EXCELLENT | 3092 |
| tasmin | rcp85 | 1.337 | 1.161 | 0.187 | 0.243 | EXCELLENT | 3092 |

Figure S15. Showing the Discontinuity analyses for MPI-LR_CDX

Table S3. Table showing Discontinuity Statistic for MPI-MR_CDX

| **Variable** | **Scenario** | **Mean**  **RMSD** | **Std**  **RMSD** | **Mean**  **NRMSD** | **Std**  **NRMSD** | **Quality** | **Grid Cell**  **Count** |
| --- | --- | --- | --- | --- | --- | --- | --- |
| pr | historical | 0.000 | 0.000 | 0.426 | 0.159 | EXCELLENT | 2390 |
| pr | rcp26 | 0.000 | 0.000 | 0.445 | 0.173 | EXCELLENT | 2390 |
| pr | rcp85 | 0.000 | 0.000 | 0.442 | 0.168 | EXCELLENT | 2390 |
| rsds | historical | 9.065 | 6.610 | 0.140 | 0.100 | EXCELLENT | 2390 |
| rsds | rcp26 | 9.020 | 6.700 | 0.137 | 0.101 | EXCELLENT | 2390 |
| rsds | rcp85 | 8.855 | 6.556 | 0.134 | 0.098 | EXCELLENT | 2390 |
| tas | historical | 1.032 | 1.319 | 0.210 | 0.454 | EXCELLENT | 2390 |
| tas | rcp26 | 1.041 | 1.325 | 0.209 | 0.444 | EXCELLENT | 2390 |
| tas | rcp85 | 1.380 | 2.734 | 0.208 | 0.363 | EXCELLENT | 2390 |
| tasmax | historical | 1.140 | 1.312 | 0.207 | 0.332 | EXCELLENT | 2390 |
| tasmax | rcp26 | 1.150 | 1.317 | 0.203 | 0.321 | EXCELLENT | 2390 |
| tasmax | rcp85 | 1.467 | 2.649 | 0.209 | 0.311 | EXCELLENT | 2390 |
| tasmin | historical | 1.145 | 1.325 | 0.202 | 0.344 | EXCELLENT | 2390 |
| tasmin | rcp26 | 1.150 | 1.333 | 0.203 | 0.343 | EXCELLENT | 2390 |
| tasmin | rcp85 | 1.395 | 2.032 | 0.208 | 0.311 | EXCELLENT | 2390 |

Figure S16. Same as Figure S15, but for ESM-MR_CDX

Table S4. Table showing Discontinuity Statistic for NorESM1_CDX

| **Variable** | **Scenario** | **Mean**  **RMSD** | **Std**  **RMSD** | **Mean**  **NRMSD** | **Std**  **NRMSD** | **Quality** | **Grid Cell**  **Count** |
| --- | --- | --- | --- | --- | --- | --- | --- |
| pr | historical | 0.000 | 0.000 | 0.421 | 0.067 | EXCELLENT | 3092 |
| pr | rcp26 | 0.000 | 0.000 | 0.425 | 0.064 | EXCELLENT | 3092 |
| pr | rcp85 | 0.000 | 0.000 | 0.433 | 0.065 | EXCELLENT | 3092 |
| rsds | historical | 14.301 | 4.913 | 0.177 | 0.065 | EXCELLENT | 3092 |
| rsds | rcp26 | 14.189 | 4.972 | 0.175 | 0.065 | EXCELLENT | 3092 |
| rsds | rcp85 | 13.987 | 4.979 | 0.172 | 0.063 | EXCELLENT | 3092 |
| tas | historical | 0.535 | 0.596 | 0.206 | 0.304 | EXCELLENT | 3092 |
| tas | rcp26 | 0.542 | 0.592 | 0.199 | 0.283 | EXCELLENT | 3092 |
| tas | rcp85 | 0.548 | 0.587 | 0.174 | 0.235 | EXCELLENT | 3092 |
| tasmax | historical | 0.738 | 0.574 | 0.220 | 0.256 | EXCELLENT | 3092 |
| tasmax | rcp26 | 0.748 | 0.570 | 0.214 | 0.238 | EXCELLENT | 3092 |
| tasmax | rcp85 | 0.759 | 0.563 | 0.200 | 0.205 | EXCELLENT | 3092 |
| tasmin | historical | 0.594 | 0.583 | 0.190 | 0.228 | EXCELLENT | 3092 |
| tasmin | rcp26 | 0.597 | 0.583 | 0.188 | 0.224 | EXCELLENT | 3092 |
| tasmin | rcp85 | 0.601 | 0.579 | 0.169 | 0.197 | EXCELLENT | 3092 |

Figure S17. Same as Figure S15, but for NorESM1_CDX

Table S5. Table showing Discontinuity Statistic for HadGEM2_CDX

| **Variable** | **Scenario** | **Mean**  **RMSD** | **Std**  **RMSD** | **Mean**  **NRMSD** | **Std**  **NRMSD** | **Quality** | **Grid Cell**  **Count** |
| --- | --- | --- | --- | --- | --- | --- | --- |
| pr | historical | 0.000 | 0.000 | 0.415 | 0.057 | EXCELLENT | 3092 |
| pr | rcp26 | 0.000 | 0.000 | 0.426 | 0.060 | EXCELLENT | 3092 |
| pr | rcp85 | 0.000 | 0.000 | 0.431 | 0.058 | EXCELLENT | 3092 |
| rsds | historical | 15.182 | 4.743 | 0.178 | 0.063 | EXCELLENT | 3092 |
| rsds | rcp26 | 14.956 | 4.699 | 0.176 | 0.062 | EXCELLENT | 3092 |
| rsds | rcp85 | 14.719 | 4.740 | 0.174 | 0.063 | EXCELLENT | 3092 |
| tas | historical | 0.530 | 0.597 | 0.205 | 0.305 | EXCELLENT | 3092 |
| tas | rcp26 | 0.537 | 0.594 | 0.201 | 0.290 | EXCELLENT | 3092 |
| tas | rcp85 | 0.548 | 0.591 | 0.157 | 0.214 | EXCELLENT | 3092 |
| tasmax | historical | 0.728 | 0.574 | 0.217 | 0.253 | EXCELLENT | 3092 |
| tasmax | rcp26 | 0.743 | 0.572 | 0.216 | 0.242 | EXCELLENT | 3092 |
| tasmax | rcp85 | 0.761 | 0.568 | 0.189 | 0.195 | EXCELLENT | 3092 |
| tasmin | historical | 0.594 | 0.583 | 0.190 | 0.229 | EXCELLENT | 3092 |
| tasmin | rcp26 | 0.599 | 0.580 | 0.189 | 0.223 | EXCELLENT | 3092 |
| tasmin | rcp85 | 0.605 | 0.580 | 0.156 | 0.180 | EXCELLENT | 3092 |

Figure S18. Same as Figure S15, but for HadGEM2_CDX

References

1	Yakubu, F., Böhner, J., Schickhoff, U., Scholten, T. & Hasson, S. u. Global Bias-Corrected CORDEX Datasets at Half Degree Resolution. *Scientific Data* **12**, 1781 (2025). <https://doi.org/10.1038/s41597-025-06200-4>

2	Taylor, K. E. Summarizing multiple aspects of model performance in a single diagram. *Journal of geophysical research: atmospheres* **106**, 7183-7192 (2001).

3	Yakubu, F., Böhner, Jürgen, Schickhoff, Udo, Scholten, Thomas, & Hasson, Shabeh Ul. Global Bias-Corrected CORDEX Dataset at Quarter Degree Resolution (Version 2025_fv0.01) [Data set]. . (2025). <https://doi.org/10.25592/uhhfdm.17799>
